# Regulation of *Arabidopsis* Flowering by the Histone Mark Readers MRG1/2 via Interaction with CONSTANS to Modulate *FT* Expression

**DOI:** 10.1371/journal.pgen.1004617

**Published:** 2014-09-11

**Authors:** Zhongyuan Bu, Yu Yu, Zepeng Li, Yanchao Liu, Wen Jiang, Ying Huang, Ai-Wu Dong

**Affiliations:** 1 State Key Laboratory of Genetic Engineering, Collaborative Innovation Center for Genetics and Development, Institute of Plant Biology, School of Life Sciences, Fudan University, Shanghai, China; 2 National Center for Protein Science Shanghai, Graduate University of Chinese Academy of Sciences, State Key Laboratory of Molecular Biology, Institute of Biochemistry and Cell Biology, Shanghai Institutes for Biological Sciences, Chinese Academy of Sciences, Shanghai, China; The University of Oklahoma, United States of America

## Abstract

Day-length is important for regulating the transition to reproductive development (flowering) in plants. In the model plant *Arabidopsis thaliana*, the transcription factor CONSTANS (CO) promotes expression of the florigen *FLOWERING LOCUS T* (*FT*), constituting a key flowering pathway under long-day photoperiods. Recent studies have revealed that *FT* expression is regulated by changes of histone modification marks of the *FT* chromatin, but the epigenetic regulators that directly interact with the CO protein have not been identified. Here, we show that the *Arabidopsis* Morf Related Gene (MRG) group proteins MRG1 and MRG2 act as H3K4me3/H3K36me3 readers and physically interact with CO to activate *FT* expression. *In vitro* binding analyses indicated that the chromodomains of MRG1 and MRG2 preferentially bind H3K4me3/H3K36me3 peptides. The *mrg1 mrg2* double mutant exhibits reduced mRNA levels of *FT*, but not of *CO*, and shows a late-flowering phenotype under the long-day but not short-day photoperiod growth conditions. MRG2 associates with the chromatin of *FT* promoter in a way dependent of both CO and H3K4me3/H3K36me3. *Vice versa*, loss of *MRG1* and *MRG2* also impairs CO binding at the *FT* promoter. Crystal structure analyses of MRG2 bound with H3K4me3/H3K36me3 peptides together with mutagenesis analysis *in planta* further demonstrated that MRG2 function relies on its H3K4me3/H3K36me3-binding activity. Collectively, our results unravel a novel chromatin regulatory mechanism, linking functions of MRG1 and MRG2 proteins, H3K4/H3K36 methylations, and CO in *FT* activation in the photoperiodic regulation of flowering time in plants.

## Author Summary

The photoperiodic flowering in *Arabidopsis* requires the key regulator CO and its target gene *FT*. However, how CO regulates *FT* expression in the context of chromatin remains largely obscure. In this work, we present *Arabidopsis* MRG1/2 as novel chromatin effectors directly involved in the CO-FT photoperiodic flowering. Firstly, MRG1/2 proteins are identified as recognition factors of H3K4 and H3K36 methylation via their chromodomains. The *mrg1 mrg2* double mutant shows a late-flowering phenotype only under long-day conditions through down-regulation of *FT* but not of *CO*. MRG2 can directly target *in vivo* the *FT* promoter chromatin in a H3K4me3/H3K36me3-level dependent manner. More importantly, MRG2 and CO physically interact and enhance each other's binding to the *FT* promoter *in planta*. Determination of co-crystal structures of MRG2 with H3K4me3/H3K36me3 peptides and mutagenesis of a key amino acid residue involved in structural interaction demonstrate that MRG2 reader activity is essential for *in planta* function. Taken together, our findings uncover a novel mechanism of *FT* activation in flowering promotion and provide a striking example of mutual interplay between a transcription factor and a histone methylation reader in transcription regulation.

## Introduction

The timing of floral transition from vegetative to reproductive development is a critical event in the plant life cycle and is coordinated by internal and environmental cues [1]–[3]. In *Arabidopsis*, the photoperiodic flowering pathway is regulated by the transcription factor CONSTANS (CO) and the florigen FLOWERING LOCUS T (FT) [4]. Circadian-clock regulated *CO* mRNA and light-dependent stabilization of CO protein are crucial for activation of *FT* expression in leaves under long days (LDs) but not short days (SDs); the FT protein is then translocated to the shoot apical meristem, where it promotes flowering [4]. The CO protein can bind to specific cis-elements in the *FT* promoter either by itself [5] or in a complex with CCAAT-binding factors [6].

Histone lysine methylation is an important epigenetic mechanism for the regulation of gene expression. Recent studies have revealed that chromatin mechanisms play important roles in flowering time by regulating the expression of key flowering-regulatory genes [7]. For example, *FT* expression is affected by several factors, including H3K27 methyltransferase CLF [8]–[10], H3K27 demethylase JMJ12/REF6 [11], H3K4 demethylases ELF6 and JMJ14/JMJ4 [12]–[14], and AFR1/2-HDAC histone deacetylase [15]. More recently, other histone methyltransferases (ATX1, ATX2, SDG8, SDG25) and demethylases (LDL1/2) were reported to affect histone methylation status at the *FT* chromatin [16].

It was proposed that chromatin with methylated histone residues can be specifically recognized by the chromatin effectors that act as readers, which might ultimately direct downstream functions [17], [18]. The *Arabidopsis* histone methylation reader proteins ORC1 [19], AtING [20], AL [20], [21], WDR5a [22], SDG8/ASHH2 [23], [24], LHP1/TFL2 [25], [26], PICKLE [27], and rice protein CHR729 [28] were found to interact with methylated H3K4 or/and H3K27, and also affect many aspects of plant development from mutant analyses. Although chromatin effectors related to several lysine residues were identified, the proteins recognizing H3K36 methylation remain unknown in plant. In addition, histone mark readers that are directly involved in CO-FT regulatory pathway have not been identified.

To identify H3K36 methylation readers, we performed an *in vitro* peptide pull-down experiment using *Arabidopsis* nuclear extracts, and identified among proteins detected with mass spectrometry the *Arabidopsis* Morf Related Gene (MRG) group protein MRG2 as a binding protein of the histone H3 N-terminal tail methylated at lysine 36. MRG1 and MRG2 belong to the MRG protein family, with highly conserved members in fungi, plants, and animals. Several family members, such as Esa1p-associated factor-3 (EAF3), MRG on chromosomes 15 (MRG15), and male-specific lethal (MSL3) have been found in yeast and animals as recognition factors of H3K36 methylation [29]–[31]. The yeast *eaf3* deletion causes no obvious growth phenotypes and only has a very modest effect on transcription [32], [33], while a mutation in the *Drosophila MSL3* gene led to male lethality [34], and overexpression of human *MRG15* results in abnormal nuclear morphologies and cell death [35], making further studies of *in vivo MRG* functions difficult in animals.

We show here using viable *Arabidopsis* mutants that MRG1/2 promote photoperiodic flowering in *Arabidopsis*. MRG1/2 proteins act as novel chromatin effectors directly involved in the CO-dependent *FT* activation in the photoperiodic flowering pathway. The *mrg1 mrg2* double mutant exhibits reduced *FT* mRNA level, with a normal *CO* mRNA level, and is late-flowering only under LDs. We further demonstrate that MRG2 and CO interact with each other physically and enhance each other's binding to the *FT* promoter region, thereby activating *FT* transcription. Furthermore, the co-crystal structures of MRG2 with H3K4me3/36me3 peptides reveal the residues important for peptide binding and a site-specific MRG2 mutation abolishes both histone mark binding activity and flowering time regulation, providing a direct link between the biochemical activity of MRG1/2 proteins and their *in vivo* biological functions.

## Results

### Identification of MRG1/2 as specific binding proteins for methylated H3K4 and H3K36

An *in vitro* peptide pull-down assay was used to identify the proteins that bind to histone H3 tail methylated at K36. Biotinylated histone H3 peptides (amino acids 21 to 44), with either unmethylated (control) or tri-methylated K36, were immobilized on the streptavidin-coated beads and incubated with *Arabidopsis* nuclear extracts. Mass spectrometry of proteins bound to the H3 peptides tri-methylated at K36 identified MRG2, which is a member of the MRG protein family. Members in this group have the same domain organization, with a chromodomain (chromatin organization modifier domain) near the N-terminus, and a MRG domain near the C-terminus (Figure S1). MRG2 has a homolog in *Arabidopsis*, MRG1, which shares with MRG2 49% identity and 65% similarity at the amino acid sequence level.

**Figure 1:**
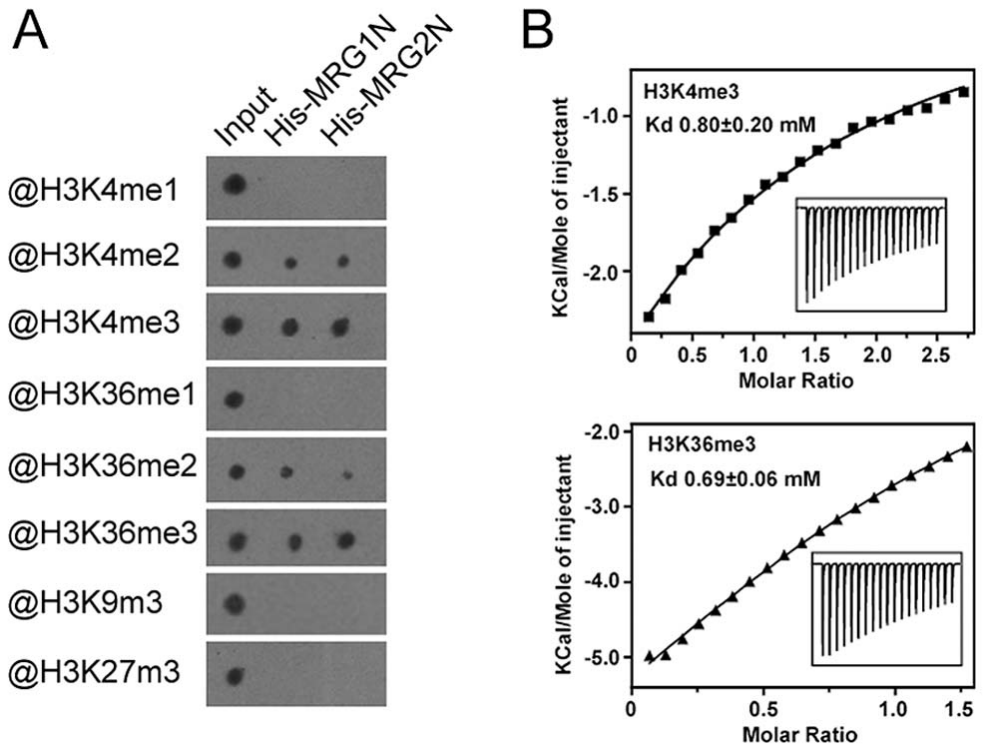
Chromodomains of MRG1 and MRG2 specifically bind to tri-methylated H3K4 and H3K36 *in vitro*. A. Binding assays of N-terminal His-tagged chromodomains of MRG1 (His-MRG1N) and MRG2 (His-MRG2N) with H3 peptides containing varying degrees of methylation at K4, K9, K27, or K36. B. ITC measurements of binding between the MRG2 chromodomain and histone peptides. Top panel, MRG2 and H3K4me3 peptide; bottom panel, MRG2 and H3K36me3 peptide.

To analyze the *in vitro* binding activity of MRG1 and MRG2, we tested whether their chromodomains could bind to H3 peptides containing different methylations at K4, K9, K27, or K36 using a pull-down assay. The chromodomains of MRG1 and MRG2 could bind to both the H3K4me2/me3 and H3K36me2/me3 peptides, but not to mono-methylated H3K4 and H3K36 peptides (Figure 1A). The two MRG proteins had a higher affinity to the tri-methylated peptides than to the di-methylated forms in the dot-blot binding assay (Figure 1A). In contrast, H3K9me3 and H3K27me3 were not recognized by either of these two proteins (Figure 1A), indicating a degree of MRG binding specificity. To further verify the specificity of the binding, an isothermal titration calorimetry (ITC) assay was performed using free-labeled histone peptides as substrates. H3K4me3 (residues 1–9) and H3K36me3 (residues 31–41) exhibited detectable binding to MRG2 chromodomain (residues 41–123) (K_d_ values: 0.8 mM for H3K4me3; 0.69 mM for H3K36me3) (Figure 1B). On the other hand, MRG2 chromodomain bound to H3K4me2 (residues 1–9), H3K36me2 (residues 31–41), H3K9me3 (residues 4–13) and H3K27me3 (residues 23–31) at a relatively low affinity (K_d_ values: 2.4 mM for H3K4me2; 2.6 mM for H3K36me2; 1.2 mM for H3K9me3; 2.5 mM for H3K27me3) (Figure S2). The ITC binding assay results were consistent with those of the *in vitro* pull-down assay, showing the binding specificity of MRG1/2 with tri-methylated H3K4 and H3K36 *in vitro*.

### MRG1/2 promote photoperiodic flowering in *Arabidopsis*

**Figure 2:**
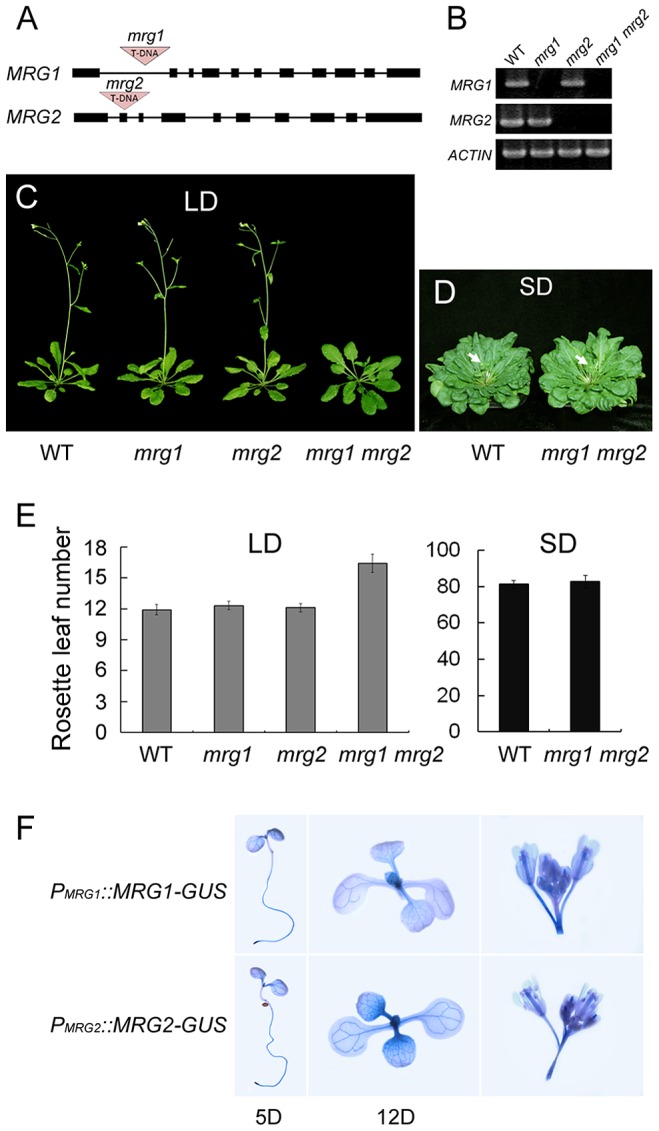
MRG1 and MRG2 act redundantly in flowering time control of *Arabidopsis* in the photoperiodic flowering pathway. A. Gene structure of *mrg1* and *mrg2* mutant alleles. Dark boxes represent exons; lines represent introns; red triangles indicate T-DNA insertions. B. RT-PCR analysis of *MRG1* and *MRG2* expression in leaves of *mrg1*, *mrg2*, and *mrg1 mrg2* plants. *ACTIN2* was used as the internal control. C. Phenotypes of the wild-type (WT), the single mutants *mrg1* and *mrg2*, and the double mutant *mrg1 mrg2* grown under long-day photoperiods (LD; 16 h light: 8 h dark). D. Phenotypes of WT and *mrg1 mrg2* plants grown under short-day photoperiods (SD; 8 h light: 16 h dark). E. Flowering time, as measured by rosette leaf number at bolting, in plants grown under LD and SD conditions. The mean value from 20 plants is shown. Error bars represent standard deviations. F. Tissue expression pattern analyses of *MRG1* and *MRG2* by histochemical GUS staining in *P_MRG1_::MRG1-GUS* and *P_MRG2_::MRG2-GUS* transgenic plants. Staining was performed at different times after seed germination (day 5 or 12), and in inflorescences.

To investigate the *in vivo* function of *MRG1/2*, the T-DNA insertional mutants *mrg1* and *mrg2*, obtained from the *Arabidopsis* Biological Resource Center (ABRC, http://www.arabidopsis.org) and the Saskatoon collection [36] (http://aafc-aac.usask.ca/FST/), were used. The single mutants of *mrg1* and *mrg2* carry T-DNA insertions in the first intron and the second exon, respectively (Figure 2A). Although *MRG1* and *MRG2* transcripts were undetectable in both mutants (Figure 2B), their overall phenotypes were normal (Figure 2C). Next, we constructed and analyzed the *mrg1 mrg2* double mutant, which showed late-flowering under long-day (LD) conditions (Figure 2C and 2E). Both *MRG1* and *MRG2* were mainly expressed in the vasculature of cotyledons and true leaves, and also in roots and inflorescences, as evidenced by histochemical GUS staining in *P_MRG1_::MRG1-GUS* and *P_MRG2_::MRG2-GUS* transgenic plants (Figure 2F). The late-flowering phenotype of *mrg1 mrg2* double mutant could be fully rescued by introducing either *P_MRG1_::MRG1-GUS* or *P_MRG2_::MRG2-GUS* into the plants, indicating that MRG1 and MRG2 are functionally redundant in the control of flowering time in *Arabidopsis*. Additionally, the *mrg1 mrg2* late-flowering phenotype was specific for LD conditions. Under short-day (SD) conditions, the double mutant showed a flowering time similar to the wild-type (Figure 2D and 2E), suggesting that the *mrg1 mrg2* double mutant is defective in the photoperiodic flowering pathway.

### MRG1/2 are required for normal activation of *FT* expression

**Figure 3:**
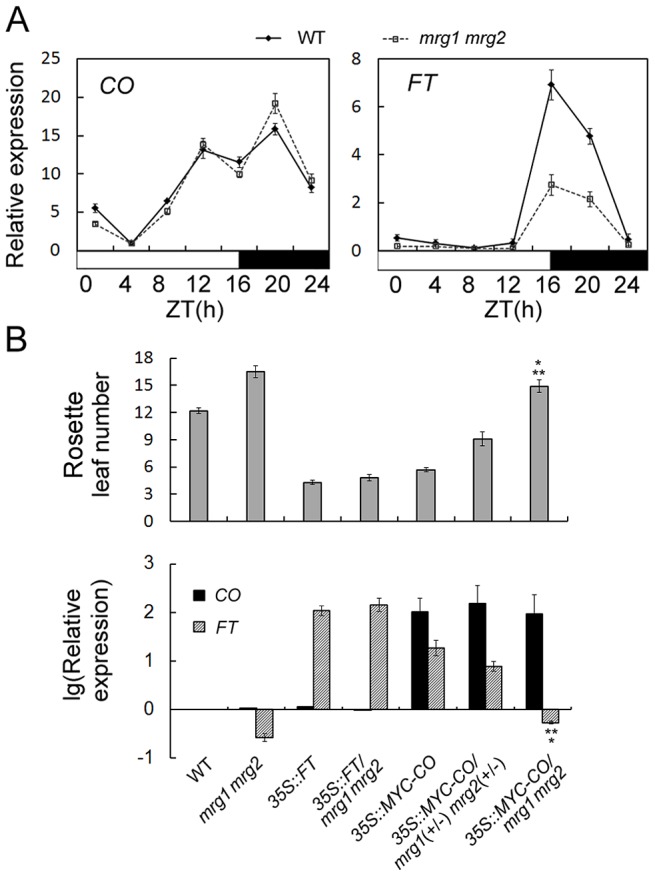
MRG1/2 are required for normal activation of *FT* expression. A. Diurnal expression of *CO* and *FT* genes in wild-type (WT) and *mrg1 mrg2* double mutant. Time is expressed as hours from dawn (ZT, zeitgeber time). Values are normalized to *ACTIN2*. Error bars show standard deviation from three biological replicates. B. Top panel, flowering time of the wild-type (WT), *mrg1 mrg2* double mutant, transgenic *35S::FT* plants in wild-type (*35S::FT*) or in *mrg1 mrg2* (*35S::FT/mrg1 mrg2*), transgenic *35S::MYC-CO* plants in wild-type (*35S::MYC-CO*), in heterozygous for *mrg1 mrg2* (*35S::MYC-CO/mrg1*(+/−) *mrg2*(+/−)), or in homozygous for *mrg1 mrg2* (*35S::MYC-CO/mrg1 mrg2*) grown in LDs. The mean value from 20 plants is shown. Error bars represent standard deviations. Bottom panel, Relative expression of *CO* and *FT* in indicated genotypes at ZT16. Values are presented as logarithmic mode (lg) of relative expression normalized to *ACTIN2*. Error bars show standard deviation from three biological replicates. A single asterisk indicates the statistically significant difference between *35S::MYC-CO/mrg1 mrg2* plants and the wild-type (P<0.05), and double asterisks indicate the statistically significant difference between *35S::MYC-CO/mrg1 mrg2* plants and the *mrg1 mrg2* double mutants (P<0.05).

To investigate the effect of MRG1/2 on flowering time in response to changes of photoperiod, we traced expression of the key genes *CO* and *FT* over a 24-h period in wild-type and *mrg1 mrg2* double mutant plants. The expression of *CO* and *FT* shows different diurnal patterns with their transcript levels [37]. Under LDs, high levels of wild-type *CO* expression are detected from late afternoon through the first half of the night, while induction of *FT* transcription occurs at the end of the day (16 h after dawn, ZT16) (Figure 3A). While the *CO* expression pattern in *mrg1 mrg2* was similar to that of the wild-type, the *FT* transcript level in *mrg1 mrg2* was lower than that of wild-type plants (Figure 3A), suggesting that MRG1 and MRG2 may be involved in activation of *FT* transcription in the photoperiodic flowering pathway.

To further test where in the photoperiodic pathway MRG1/2 act, we introduced *35S::FT* and *35S::MYC-CO* constructs into the late-flowering *mrg1 mrg2* double mutant through introgression of the transgenes. Transgenic plants overexpressing *FT* or *CO* in the wild-type flowered significantly earlier (Figure 3B) [37], [38]. Overexpression of *FT* fully rescued the late-flowering phenotype of the *mrg1 mrg2* double mutant (Figure 3B), indicating that *MRG1/2* indeed act upstream of *FT*. In contrast, overexpression of *CO* failed to induce *FT* transcription in *mrg1 mrg2* background, and the plants still displayed late-flowering phenotype comparing with the wild-type plants, although these *35S::MYC-CO/mrg1 mrg2* plants showed slightly higher *FT* expression and slightly earlier flowering time comparing with the *mrg1 mrg2* double mutant (Figure 3B). It's to be noted that the significantly increased expression levels of *CO* (around 100 folds of that in the wild-type) were similar in the *35S::MYC-CO* transgenic plants in wild-type (2 copies of *35S::MYC-CO*), heterozygous (1 copy of *35S::MYC-CO*) or homozygous *mrg1 mrg2* (2 copies of *35S::MYC-CO*) backgrounds. To further verify this phenotype, another transgene *35S::FLAG-CO* was also constructed and introduced into the *mrg1 mrg2* double mutant. As shown in Figure S3, transgenic plants overexpressing *FLAG-CO* in the wild-type exhibited high level of *FT* transcription and early-flowering phenotype. However, similar to *35S::MYC-CO/mrg1 mrg2*, overexpression of *FLAG-CO* also failed to induce *FT* transcription in *mrg1 mrg2* background (with similar expression level of *CO* as that in the wild-type background, Figure S3) and the plants still flowered a little bit later than the wild-type, suggesting that the CO function in promoting flowering requires MRG1/2 proteins. When constructing *35S::MYC-CO/mrg1 mrg2* plants, we found that the *35S::MYC-CO* transgenic plants in wild-type, heterozygous (1 copy of *MRG1* and 1 copy of *MRG2*) or homozygous *mrg1 mrg2* backgrounds exhibited different *FT* transcription levels and flowering time (Figure 3B), noting that the plants with either 1 copy or 2 copies of *35S::MYC-CO* showed similar expression levels of *CO*. The heterozygous *35S::MYC-CO*/*mrg1(+/−) mrg2(+/−)* plants, with about 30% reduction of *MRG1* or *MRG2* (Figure S4), showed *FT* activation and earlier flowering comparing with the wild-type plants, but their flowering time was still later than that of the *35S::MYC-CO* plants in wild-type background, indicating that the requirement of MRG1/2 in the effect of CO overexpression is in a dosage-dependent manner. These results imply an important role for MRG1/2 in the photoperiod flowering pathway in *Arabidopsis*, and MRG1/2 are involved in the *FT* transcriptional activation.

### MRG2 binds the chromatin at the *FT* promoter

**Figure 4:**
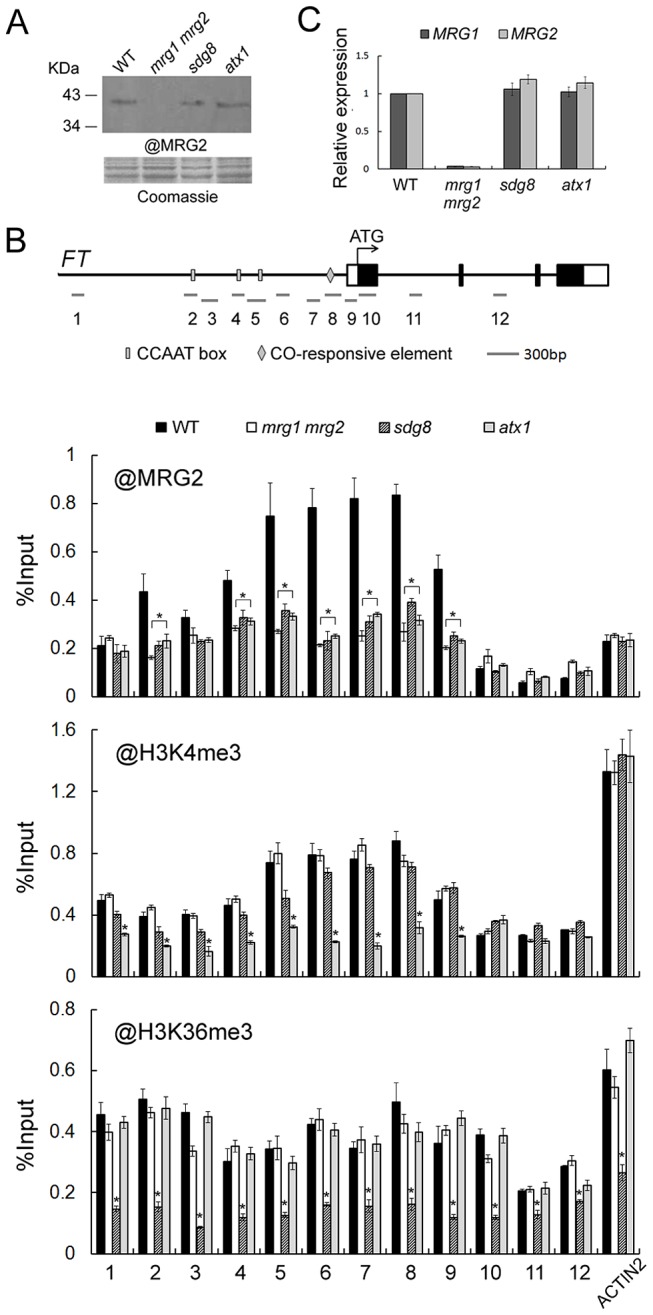
MRG2 binds the chromatin at the *FT* promoter. A. Protein levels of MRG2 in WT, *mrg1 mrg2*, *sdg8*, and *atx1* plants at ZT16 verified by the antibody against MRG2. B. ChIP analysis using @MRG2, @H3K4me3 or @H3K36me3 at *FT* chromatin in WT, *mrg1 mrg2*, *sdg8*, and *atx1* plants at ZT16. Error bars show standard deviation from three biological replicates. Asterisks indicate statistically significant differences between the indicated genotypes and the wild-type (P<0.01). C. Relative expression levels of *MRG1* and *MRG2* in indicated genotypes at ZT16. Values are normalized to *ACTIN2*. Error bars show standard deviation from three biological replicates.

To obtain evidence for the molecular mechanisms of regulation of *FT* by MRG1/2 proteins, we performed a Chromatin Immuno-Precipitation (ChIP) assay to analyze possible *in vivo* binding of MRG1/2 proteins to the *FT* locus. The antibody against MRG2 (Figure 4A) was used in ChIP assays in wild-type and *mrg1 mrg2* plants. The endogenous MRG2 protein was obviously enriched in the *FT* promoter regions in wild-type comparing with that in *mrg1 mrg2* double mutant (Figure 4B), indicating that the MRG2 protein targets directly to the *FT* promoter. To test whether MRG2 binding to the *FT* promoter region was dependent on the H3K4 and H3K36 methylation status of the chromatin, we used loss-of-function mutants *atx1* and *sdg8*, which affect, respectively, the *ATX1* gene encoding a H3K4 methyltransferase and the *SDG8* gene encoding a H3K36 methyltransferase [39], [40]. As a control, the *atx1* and *sdg8* mutations did not affect the expression levels of *MRG1/2* (Figure 4A and 4C). ChIP analysis indicated that *atx1* and *sdg8* mutants showed reduced H3K4me3 and H3K36me3 levels, respectively, in most *FT* chromatin regions, when compared with wild-type plants (Figure 4B). A detectable reduced H3K4me3 levels in some *FT* promoter regions were observed in *sdg8*, while the H3K36me3 levels of *FT* chromatin were not affected in *atx1* comparing with wild-type. The overall H3K4me3 and H3K36me3 levels of *FT* in *mrg1 mrg2* double mutant were similar as those in the wild-type plants, indicating that the deletions of *MRG1* and *MRG2* do not change the H3K4me3 and H3K36me3 levels of *FT* chromatin. Along with reduced H3K4me3 and H3K36me3 levels, MRG2 enrichment at the *FT* promoter in *atx1* and *sdg8* mutants was decreased (Figure 4B), suggesting that MRG2 may directly bind to the *FT* promoter via recognition of methylated H3K4 and H3K36 *in planta*.

### MRG2 and CO interact physically and enhance each other's binding to the *FT* promoter

**Figure 5:**
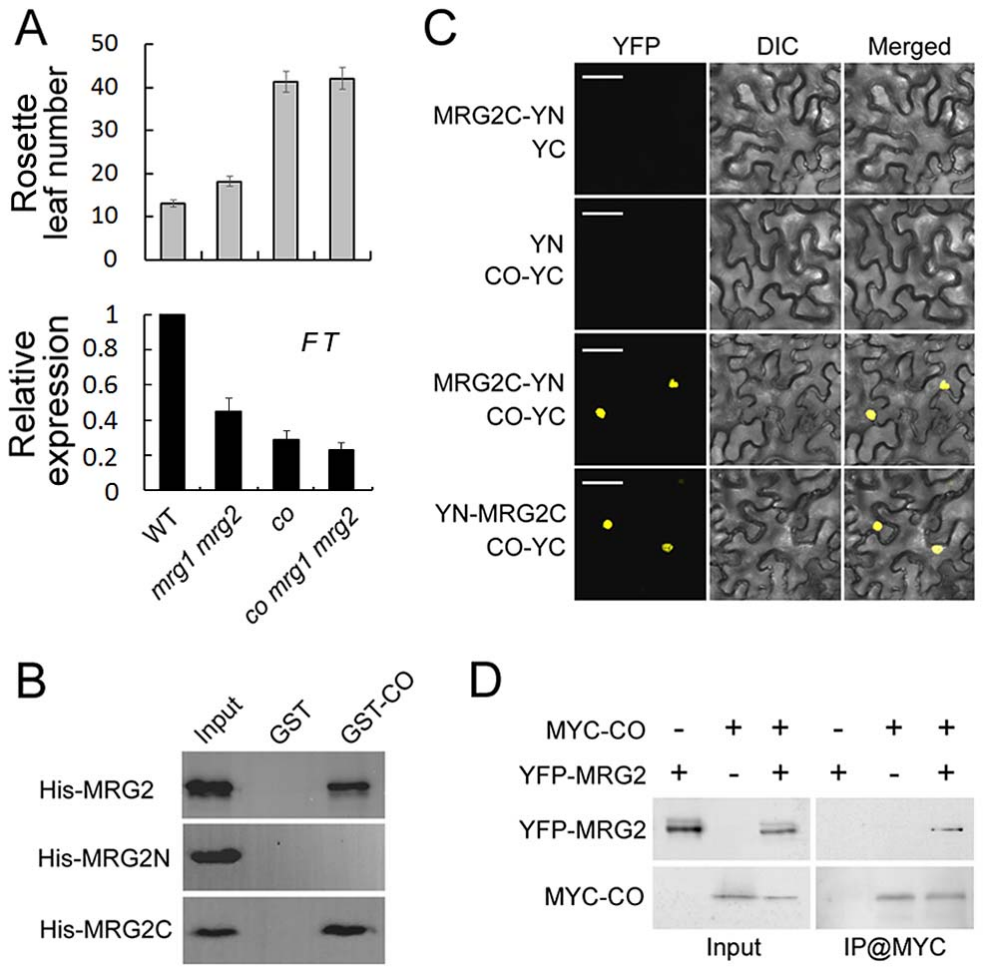
MRG2 physically interacts with CO. A. Top panel, flowering time of WT, *mrg1 mrg2*, *co* and *co mrg1 mrg2*, as measured by rosette leaf number at bolting, in plants grown under LD conditions. The mean value from 20 plants is shown. Error bars represent standard deviations. Bottom panel, relative expression of *FT* in indicated genotypes at ZT16. Values are normalized to *ACTIN2*. Error bars show standard deviation from three biological replicates. B. Interaction of purified His-tagged MRG2 and GST-fused CO from *E. coli* by pull-down assays. C. BiFC analysis of the interaction between MRG2 and CO in tobacco leaf cells. Bar  = 50 µm. D. Co-IP detection of MRG2 and CO interaction *in planta*. Total protein extracts from plants expressing YFP-MRG2 alone, MYC-CO alone, or both YFP-MRG2 and MYC-CO were immunoprecipitated with anti-MYC affinity beads and the resulting fractions analyzed by western blot using anti-GFP antibodies (top panel) or HRP-conjugated anti-MYC monoclonal antibodies (bottom panel).

The *FT* promoter contains several canonical CCAAT boxes and one CO-responsive element (CORE), these are essential for CO-mediated *FT* activation. Given that the CCAAT boxes and CORE sequence are within the MRG2-enriched regions of the *FT* promoter, we hypothesized that MRG1/2 proteins might be involved in the CO-dependent regulation of *FT* expression. To test this hypothesis, firstly we introduced a *co* mutant into the *mrg1 mrg2* double mutant and found that the *co mrg1 mrg2* triple mutant flowered at nearly the same time as *co* in LDs (Figure 5A), suggesting that MRG1/2 may act to regulate *FT* in a CO-dependent genetic pathway.

The facts that MRG1/2 and CO are both positive regulators of *FT* expression, that they both express mainly in the vasculature of leaves, and that they bind to the overlapped regions of *FT* promoter ([41] and this study) drive us to test whether MRG1/2 might interact with CO physically. To test this hypothesis, we examined the protein-protein interaction between MRG2 and CO. In an *in vitro* pull-down assay, we found that GST-fused CO but not GST alone could pull-down MRG2 tagged with multiple His (histidine) residues (Figure 5B). To determine which domain in MRG2 was required for this interaction, we performed a truncation analysis and found that the MRG domain (MRG2C) but not the chromodomain (MRG2N) of MRG2 contributed to binding to CO (Figure 5B). To verify the interaction observed in the pull-down assay, an *in vivo* binding assay was performed using bimolecular fluorescence complementation (BiFC) analysis. When tobacco leaves were infiltrated with *Agrobacterium* cells carrying appropriate constructs, MRG2/MRG2C and CO proteins tagged with split YFP interacted *in vivo* to reconstitute the whole YFP protein with fluorescent signal; therefore only the interaction of MRG2C and CO is shown in Figure 5C. Further support for a physical interaction between MRG2 and CO *in planta* came from a co-immunoprecipitation (CoIP) experiment, in which YFP-MRG2 was detected in the MYC-CO immunoprecipitated fraction from transgenic *Arabidopsis* expressing both MYC-CO and YFP-MRG2 (Figure 5D).

**Figure 6:**
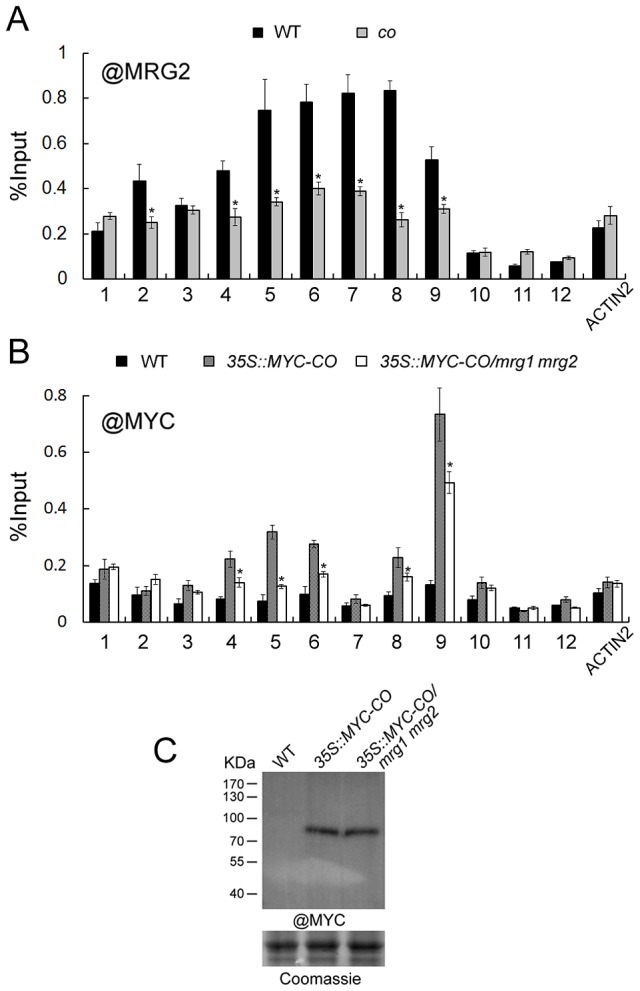
MRG2 and CO enhance each other's binding to the *FT* promoter. A. Enrichment of MRG2 at the *FT* promoter at ZT16 in wild-type and *co* mutant at ZT16. Error bars show standard deviation from three biological replicates. Asterisks indicate statistically significant differences between *co* and the wild-type (P<0.01). B. Enrichment of MYC-CO at the *FT* promoter at ZT16 in indicated genotypes at ZT16. Error bars show standard deviation from three biological replicates. Asterisks indicate statistically significant differences between *35S::MYC-CO/mrg1 mrg2* plants and *35S::MYC-CO* plants (P<0.01). C. Protein levels of MYC-tagged CO in indicated genotypes at ZT16.

To explore the role of CO and MRG1/2 protein-protein interaction at the *FT* promoter, we examined MRG2 binding at *FT* upon loss of CO function by a ChIP assay using the anti-MRG2 antibody in the *co-1* mutant. Loss of CO function resulted in declined MRG2 binding to the *FT* promoter (Figure 6A) without changes of the *MRG1/2* transcription levels (Figure S5A) and H3K4me3/H3K36me3 levels in *FT* promoter (Figure S5B), indicating that CO is required for normal levels of MRG2 binding to the *FT* chromatin, and supporting the genetic result that the MRG1/2 function in promoting flowering depends on CO (Figure 5A). Conversely, to test whether MRG1/2 proteins affect the level of CO at the *FT* promoter, *35S::MYC-CO/mrg1 mrg2* plants were used for a ChIP analysis of the MYC-CO protein with anti-MYC antibody. As observed previously [41], CO peaks near the *FT* transcription start site where the CORE sequence is located (Figure 6B). In addition, upstream of this CO peak, there is a relatively low but detectable enrichment of CO at the *FT* promoter (Figure 6B), where several canonical CCAAT boxes are embedded. Western blot analysis of total protein extracts revealed that CO protein accumulated at similar levels in wild-type and the *mrg1 mrg2* double mutant (Figure 6C). Surprisingly, CO enrichment at the *FT* promoter decreased in *mrg1 mrg2* (Figure 6B), implying that MRG1/2 proteins may interact with CO at the *FT* promoter, and affect the stable binding of CO at the locus. This result is also consistent with that the overexpression of *CO* failed to rescue the *mrg1 mrg2* double mutant late-flowering phenotype (Figure 3B). Taken together, both our biochemical and genetic analyses demonstrate that MRG2 and CO physically interact and depend on each other for their stable association with the *FT* promoter chromatin to promote flowering.

### Co-crystal structures of MRG2 chromodomain and methylated H3 peptides reveal the residues for recognition of H3K4me3 or H3K36me3

**Figure 7:**
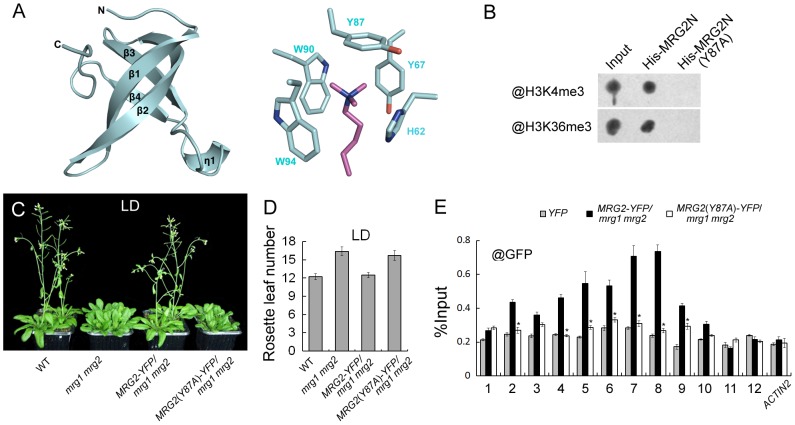
MRG1/2 binding to histone marks is required for their function in regulating flowering. A. Structure of the MRG2 chromodomain in complex with H3K36me3. The overall structure of the MRG2 chromodomain consisting of four β strands is shown in the left panel. The tri-methylated lysine sticking in the aromatic cage was surrounded by five conserved residues (right panel). Tri-methylated lysine is shown in purple; residues forming the aromatic cage are shown in cyan. B. Binding assays of His-tagged chromodomains of MRG2 (His-MRG2N) and a Y87A substitution in MRG2 chromodomain (His-MRG2N(Y87A)) with H3K4me3 and H3K36me3. C. Phenotypes of wild-type (WT), *mrg1 mrg2*, *P_MRG2_::MRG2-YFP*/*mrg1 mrg2* (*MRG2-YFP*/*mrg1 mrg2*), and *P_MRG2_::MRG2(Y87A)-YFP*/*mrg1 mrg2* (*MRG2(Y87A)-YFP/mrg1 mrg2*) plants grown under long-day photoperiods (LD; 16 h light: 8 h dark), noting that the *mrg1 mrg2* double mutants have bolted. D. Flowering time of indicated genotypes, as measured by rosette leaf number at bolting, in plants grown under LD conditions. The mean value from 20 plants is shown. Error bars represent standard deviations. E. ChIP analysis using @GFP at *FT* chromatin in indicated genotypes at ZT16. Error bars show standard deviation from three biological replicates. Asterisks indicate statistically significant differences between *MRG2(Y87A)-YFP/mrg1 mrg2* plants and *MRG2-YFP*/*mrg1 mrg2* plants (P<0.01).

To obtain the information regarding the specific MRG2 residues that are important for recognition of the histone marks H3K4me3 and H3K36me3, we determined the co-crystal structure of MRG2 and the methylated H3 peptides. To identify the suitable length of MRG2 chromodomain-containing fragments for crystallization, limited proteolysis was performed to remove unstructured flexible regions, because these tend to be difficult to crystalize. The proteolyzed samples were analyzed using mass spectrometry (MS) analysis to determine the protein sequence, resulting in the identification of a region containing the chromodomain with residues 53–123 of MRG2 as suitable for crystallization. MRG2 complexes with the H3K4me3 and H3K36me3 peptides were obtained, but the complexes with the H3K27me3 and H3K9me3 peptides could not form crystals under the same conditions, consistent with the ITC results (Figure S2). The crystals of MRG2 with H3K4me3 or H3K36me3 were diffracted to 1.68 and 1.65 Å, respectively (Table S1). The structures were solved by the molecular replacement method using the chromodomain of human MRG15 (PDB entry 2F5K) as template. In both structures, the MRG2 chromodomain contains mainly four beta-strands. However, the predicted alpha-helix located at the C-terminus is missing in the electron density map (Figure S1A and 7A). For the complexes of the MRG2 chromodomain with the H3K4me3/H3K36me3 peptides, the recognition modes are almost the same; therefore, only the complex of the MRG2 chromodomain with H3K36me3 peptide is shown in Figure 7A. The tri-methylated lysine projects into the aromatic cage formed by the His62, Tyr67, Tyr87, Trp90, and Trp94 residues of the MRG2 chromodomain (Figure 7A). The co-crystal structures clearly indicate that the chromodomain of MRG2 can directly bind to tri-methylated H3K4 and H3K36, and provide strong evidence that the aromatic cage of the MRG2 chromodomain and the associated residues are essential for the binding, the same as other chromodomain proteins [29].

### MRG1/2 binding to histone marks is required for their biological function *in vivo*

According to the co-crystal structures described above, we specifically replaced Tyr87 with Ala. Consistent with the structure, the Tyr87Ala (Y87A) mutation in MRG2 disrupted its association with H3K4me3 and H3K36me3 peptides (Figure 7B), supporting the idea that the hydrophobic pocket formed by the aromatic residues is essential for the binding, as with other chromodomain proteins [29].

To further test whether the Tyr87 residue is important for *in vivo MRG2* function, we constructed *P_MRG2_::MRG2-YFP* and *P_MRG2_::MRG2*(*Y87A*)-*YFP* fusions and introduced them into the *mrg1 mrg2* double mutant. As expected, the late-flowering phenotype of the double mutant could be fully rescued by the *P_MRG2_::MRG2-YFP* transgene (Figure 7C and 7D). On the other hand, the *P_MRG2_::MRG2*(*Y87A*)-*YFP* transgene could not rescue the *mrg1 mrg2* mutant phenotype (Figure 7C and 7D), suggesting that the capability of the MRG2 protein to bind the methylated histones is essential for its biological function *in vivo*.

We then used the *P_MRG2_::MRG2-YFP*/*mrg1 mrg2* and *P_MRG2_::MRG2*(*Y87A*)-*YFP*/*mrg1 mrg2* transgenic plants with similar mRNA and protein expression levels of *MRG2-YFP* and *MRG2*(*Y87A*)-*YFP* (Figure S6) for ChIP assays using a specific antibody against GFP. At the *FT* promoter, the MRG2-YFP proteins in the *P_MRG2_::MRG2-YFP*/*mrg1 mrg2* plants showed a similar enrichment pattern to the endogenous MRG2 protein (Figure 7E and 4B). However, in the *P_MRG2_::MRG2*(*Y87A*)-*YFP*/*mrg1 mrg2* plants (Figure 7E), in which the mutated MRG2 protein failed to bind to the tri-methylated H3K4 or H3K36 (Figure 7B), the enrichment of MRG2(Y87A)-YFP proteins to *FT* were obviously decreased, showing that the association of MRG2 with *FT* promoter depends on its H3K4me3/H3K36me3 binding activity, thus providing a direct link between the biochemical activity of MRG1/2 and their *in vivo* biological functions.

## Discussion

The photoperiodic regulation of flowering is widely observed among flowering plants. In *Arabidopsis*, this pathway requires the key regulator CO and its target gene *FT*. Although some chromatin modifiers have been reported to be involved in *FT* regulation, little is known regarding direct interaction between chromatin effectors and the CO-FT pathway. In this work, we present *Arabidopsis* histone mark readers MRG1/2 as novel chromatin effectors that interact with both the CO protein and the *FT* promoter, thus providing a chromatin regulatory mechanism linking the transcription factor CO and the H3K4/36-methylation readers MRG1/2 in *FT* activation to promote plant flowering under long-day photoperiods.

Firstly, via their chromodomain, MRG1/2 proteins act as readers that recognize methylated H3K4 and H3K36. Unlike EAF3, which bind to H3K36me3/2 and only very weakly to H3K4me3/2 [29], our *in vitro* binding and crystal structure data revealed that the chromodomain of MRG2 has a preference to interact with tri-methylated forms of both H3K4 and H3K36. The ChIP assays revealed that MRG2 binds to the *FT* promoter at positions where the tri-methylated H3K4 is enriched (Figure 4B). Loss of *ATX1* function resulted in a decrease of MRG2 enrichment at the *FT* promoter without change of *MRG1/2* expression levels (Figure 4C). Unlike H3K4me3 distribution, which associated with MRG2 binding pattern, we found that H3K36me3 was homogeneously distributed along *FT* promoter (Figure 4B). The *sdg8* mutant exhibited slightly decreased levels of H3K4me3, and significant reductions of H3K36me3 and MRG2 enrichment in most *FT* chromatin regions also with no change of *MRG1/2* expression (Figure 4C). Given that the slightly reduced H3K4me3 level in *sdg8* might not be sufficient for a severe decrease of MRG binding ability, H3K36me3 is very likely to contribute in the MRG binding *in vivo*. The *co* mutant again showed declined MRG2 binding to the *FT* promoter (Figure 6A) without changes of the *MRG1/2* transcription levels (Figure S5A). Therefore, we propose that H3K4me3/H3K36me3 and transcription factor CO may play together to specify the MRG2 enrichment in *FT* chromatin *in planta*.

The binding capability of the MRG1/2 proteins with H3K4me3/H3K36me3 is essential for their biological function *in planta* because the Y87A substitution in the chromodomain of MRG2, which lost the H3K4me3/H3K36me3 binding capacity, failed to rescue the *mrg1 mrg2* double mutant late-flowering phenotype (Figure 7). However, kinetic analysis *in vitro* revealed that MRG2 binding affinities with H3K4me3 and H3K36me3 are fairly weak (Figure 1B). Therefore, MRG2 chromodomain itself may have difficulty in remaining associated with methylated histone tails without the assistance of other factors. Here, participation of another domain, namely the MRG domain, provides extra binding affinity and specificity to exert biological function. We demonstrated that MRG2 interacts with CO via the MRG domain, and that this interaction is required for stable binding of MRG2 to H3K4me3 and H3K36me3 at the *FT* promoter, as loss of CO function results in reduction of MRG2 enrichment at the *FT* locus.

On the other hand, although the effect of *mrg1 mrg2* double mutant on flowering time in LDs is comparably mild, MRG binding to *FT* is important for CO-dependent *FT* activation because in *mrg1 mrg2* background, overexpression of *MYC-CO* (Figure 3B) or *FLAG-CO* (Figure S3) failed to induce *FT* activation, and the transgenic plants still displayed late-flowering phenotype comparing with the wild type plants. Furthermore, the requirement of MRG proteins for *CO* overexpression is in a dosage-dependent manner (Figure 3B). As to the mild late flowering phenotype of *mrg1 mrg2* double mutant, we speculate that there might be redundant factors to help CO to induce *FT* transcription, for in *mrg1 mrg2* double mutant, *FT* expression is down-regulated but not abolished. Overexpression of *MRG2* by introducing *35S::MRG2* into the wild-type plants do not affect their flowering time, indicating that the increased MRG2 is not sufficient to induce early flowering and the endogenous MRG proteins are enough. Now we do not know the reason why the deletion of *MRGs* repressed the effect of *CO* overexpression, and one possibility may be that without MRG proteins, an more effective combination of CO at *FT* chromatin probably could not be established to induce a major change in *FT* activation. CO protein is stabilized towards the end of LDs, and its abundance declines rapidly in the dark [4], [42]. MRG1/2 do not seem to affect the stability of CO proteins since they accumulate at similar levels in both wild-type and the *mrg1 mrg2* double mutant at ZT16 (Figure 6C). Therefore, we propose that MRG1/2 are critical for stabilizing CO recruitment at the *FT* locus. This reinforcement mechanism may resemble the role of H3K4me3 binding by TAF3 in the TFIID complex, in which TAF3 acts as a transcriptional coactivator of the basal transcription factor TFIID in a PHD finger-dependent manner [43].

In summary, our findings strongly support a model in which MRG1/2 proteins interact with CO to activate *FT* transcription. CO directly binds to the *FT* promoter, enhancing the recruitment of MRG1/2 proteins to the *FT* locus. In addition, MRG1/2 proteins bind to chromatin that contains tri-methylated H3K4 and H3K36 via their chromodomains, the bound MRG1/2 in turn stabilize the binding of CO and ultimately controls the activation of *FT* transcription. Therefore, MRG1/2 act as a novel type of chromatin modulators, linking H3K4/H3K36 methylations and CO in *FT* activation in the photoperiodic flowering regulation in plants.

How *FT* expression is modulated after CO and MRG1/2 binding to trigger correct flowering transition remains to be investigated. Evidence from yeast and animals shows that EAF3 and MRG15 are present in both histone acetyltransferase (HAT) and deacetylase (HDAC) complexes, and are involved in the regulation of chromatin structure [32], [44], [45]. It is therefore possible that transcriptional activation of *FT* might be due to the recruitment of subsequent chromatin effectors, such as the HAT complex. Another important issue is how *FT* expression is rhythmically regulated. Light signaling regulates CO protein stability and acts to generate daily rhythms in CO abundance [4], [42]. We propose that the association of CO with MRG1/2 may help its re-association with the *FT* promoter as MRG1/2 might be more stably bound to *FT* due to the overall stability of H3K4me3 and H3K36me3. Gu et al. revealed a periodic histone deacetylation mechanism for dampening *FT* mRNA expression at dusk, thereby modulating day length-dependent *FT* expression [15]. Further exploration will help to clarify the chromatin mechanism involved in the photoperiodic regulation of flowering time control.

## Materials and Methods

### Dot-blot binding assay

For the dot-blot binding assay, *MRG1N* (1–321, AT4G37280) and *MRG2N* (1–369, AT1G02740) cDNA were amplified by primers MRG1-1/MRG1-2 and MRG2-1/MRG2-2, respectively, and cloned into a pET-30a vector (Novagen, www.novagen.com). Site mutation was generated using a Takara MutanBEST Kit (http://www.takara-bio.com) with primers MRG2-3/MRG2-4. The *in vitro* binding assay of the chromodomain containing proteins with the histone peptides were performed as described previously [46]. Methylated H3K4 (H3K4me1/2/3, residues 1–21), H3K9 (H3K9me1/2/3, residues 1–21), H3K27 (H3K27me1/2/3, residues 21–44), and H3K36 (H3K36me1/2/3, residues 21–44) peptides were synthesized by Scilight Biotechnology (http://www.scilight-peptide.com). Anti-monomethyl-H3K4 (07-436), anti-dimethyl-H3K4 (07-030), anti-trimethyl-H3K4 (07-473), anti-trimethyl-H3K9 (07-442), and anti-trimethyl-H3K27 (07-449) were purchased from Millipore (http://www.millipore.com), and anti-monomethyl-H3K36 (ab9048), anti-dimethyl-H3K36 (ab9049), and anti-trimethyl-H3K36 (ab9050) were purchased from Abcam (http://www.abcam.com).

### Isothermal titration calorimetry (ITC)

ITC experiments were carried out at 25°C on a MicroCal iTC200 (GE Healthcare, www.gelifesciences.com). Protein and peptide were kept in an identical buffer of 50 mM Tris pH 8.0, 100 mM NaCl. The sample cell was filled with a 0.2–0.4 mM solution of protein, and peptide (2–4 mM) was added sequentially in 2 µl aliquots (total of 20 injections) at 2.5 min intervals. Binding isotherms were analyzed by fitting data into the one-site model using the ITC data analysis module Origin 7.0.

### Plant materials and growth conditions

All *Arabidopsis* alleles were derived from the Columbia ecotype. *mrg1* and *mrg2* alleles, corresponding to SALK_057762 and SK28487 T-DNA insertion lines respectively, were obtained from the Arabidopsis Biological Resource Center (ABRC, http://www.arabidopsis.org) and the Saskatoon collection [36] (http://aafc-aac.usask.ca/FST/). The *mrg1 mrg2* double mutant was created in our laboratory by genetic crossing. The mutants *sdg8-2*, *atx1-2*, and *co-1* have been previously described [39], [40], [47], as have the *35S::FT* and *35S::MYC-CO* lines [38], [48]. Higher order combinations of mutants were produced by genetic crossing. *In vitro* plant culture was performed on agar-solidified MS medium M0255 (Duchefa, http://www.duchefa.com) supplemented with 1% sucrose and 0.9% agar. The studied photoperiods were 16 h light and 8 h dark for long-day (LD), and 8 h light and 16 h dark for short-day (SD).

### Complementation of mutant plants and histochemical GUS activity assay

For GUS staining, *MRG1* and *MRG2* genomic sequences were amplified by primers MRG1-3/MRG1-4 and MRG2-5/MRG2-6, respectively. They were fused to the *GUS* genomic sequence, and then cloned into pCAMBIA1300 (CAMBIA, http://www.cambia.org). The resulting constructs of *P_MRG1_::MRG1-GUS* and *P_MRG2_::MRG2-GUS* were used to transform the *mrg1 mrg2* plants. GUS activity was assayed by incubating plant tissues in GUS staining buffer [49] for 3–6 hours at 37°C. Plant material was cleared in 70% ethanol, and observed directly under a dissecting microscope (MZ10F, Leica, Germany).

### Gene expression analysis

Total RNA was prepared from plant tissues using TRI Reagent according to the manufacturer's instructions (Invitrogen, http://www.invitrogen.com). Reverse transcription was performed using standard procedures with Improm-II reverse transcriptase (Promega, http://www.promega.com). PCR amplification from the cDNA template was performed using gene-specific primers (see Table S2). *ACTIN2* was used as a reference gene to normalize the data.

### Construction of MRG–YFP/YFP-MRG fusions and plant transformation

*MRG2* genomic sequence, amplified by primers MRG2-5/MRG2-6 and fused with *EYFP* cDNA, was cloned into the pCAMBIA1300 vector. Site mutation was generated using a Takara MutanBEST Kit (http://www.takara-bio.com) with primers MRG2-7/MRG2-8. The resulting constructs *P_MRG2_::MRG2-YFP* and *P_MRG2_::MRG2*(*Y87A*)-*YFP* were used to transform the *mrg1 mrg2* plants.

*MRG2* cDNA, amplified by primers MRG2-1/MRG2-10 and fused with *EYFP* cDNA, was cloned into the pER8 vector [50], downstream to an estrogen inducible promoter, and the construct of *pER8::YFP-MRG2* was transformed into the wild-type *Arabidopsis*.

### Chromatin Immunoprecipitation (ChIP)

ChIP was performed as previously described [39] with minor modifications. After fixation, 14-day-old seedlings were ground crudely in liquid nitrogen. Low and high salt wash buffers were supplemented with 0.1% Triton X-100. Antibodies used in this study were anti-GFP (A-11122, Invitrogen, http://www.invitrogen.com), anti-MYC (11667149001, Roche, http://www.roche-applied-science.com), anti-trimethyl-H3K4 (07-473, Millipore, http://www.millipore.com), and anti-trimethyl-H3K36 (ab9050, Abcam, http://www.abcam.com). The polyclonal antibody against MRG2 was produced by Abmart (http://www.abmart.com.cn). Quantitative real-time PCR was performed with a kit from Takara (http://www.takara-bio.com) to determine the enrichment of DNA immunoprecipitated in the ChIP experiments, using gene-specific primers listed in Table S2. The efficiency values are the ratios determined by taking a fixed aliquot of the DNA extracted from the immunoprecipitated samples and the Input. Error bars show standard deviation from three paralleled biological replicates.

### Pulldown assay

*MRG2* and *MRG2C* (442–984) cDNA were amplified with primers MRG2-1/MRG2-10 and MRG2-9/MRG2-10, respectively, and then cloned into pET-30a vector (Novagen, www.novagen.com). The resulting constructs and pET-30a-MRG2N were used for purification of His-tagged MRG2, MRG2C, and MRG2N proteins. *CO* cDNA was amplified using primers CO-1/CO-2, and then cloned into pGEX-4T-1 vector for purification of GST-fused CO proteins. Pulldown experiments were performed according to a previously described protocol [51].

### Bimolecular fluorescence complementation (BiFC) assay

BiFC was performed as described by Sun et al. [52]. For BiFC assays, *MRG2C* cDNA was amplified using primers MRG2-11/MRG2-12, and then cloned into pXY103 and pXY106 vectors [52]. *CO* cDNA was amplified by primers CO-3/CO-4, and then cloned into a pXY104 vector. Leaves of 4- to 8-week-old *Nicotiana benthamiana* plants were co-infiltrated with *Agrobacterium tumefaciens* strain GV1301 carrying transgene constructs. Localization of BiFC fluorescence was observed 2–3 days after infiltration using a confocal laser scanning microscope (LSM 710, ZEISS, Germany).

### Co-immunoprecipitation (Co-IP) assays

14-day-old seedlings expressing *YFP-MRG2* (*pER8::YFP-MRG2*/WT), *MYC-CO* (*35S::MYC-CO*/WT), or both *YFP-MRG2* and *MYC-CO* were grown on MS medium with 4 µm estrogen to induce the overexpression of YFP-MRG2, and a Co-IP assay was performed as described previously [21]. IP was performed using anti-MYC affinity gel (E6654, Sigma, http://www.sigmaaldrich.com). Immunoprecipitated proteins and the input fractions were separated on a 10% SDS-PAGE and detected by western blotting using anti-GFP antibodies (A-11122, Invitrogen, http://www.invitrogen.com) or HRP-conjugated anti-MYC monoclonal antibodies (11814150001, Roche, http://www.roche-applied-science.com).

### Crystallization and structure determination

The cDNA encoding the chromodomain of *Arabidopsis thaliana* MRG2 (residues 41–123 and 53–123) were amplified by PCR, and cloned into a pET28-SMT3 vector [53]. Following purification and removal of the tag, target proteins were concentrated to 20 mg/ml for structural and biochemical studies. To optimize the construct for crystallization, limited proteolysis was performed, and samples treated with endoproteinase Glu-C gave a single band on SDS-PAGE; the digested fragment was identified as residues from 41 to 108 by Mass Spectrometry.

A solution of 12 mg/ml MRG2 chromodomain (residues 41–123) was incubated for 2 hours with H3K36me3 peptide and endoproteinase Glu-C at a 200∶400∶1 molar ratio before crystallization. MRG2 chromodomain (residues 53–123) was incubated with H3K4me3/H3K36me3 peptide in the same way, with the exception of the Glu-C treatment. H3K4me3 and H3K36me3 peptides used for crystallization were synthesized, and indicated as residues 1–9 and 31–41, respectively. Crystallization was performed using the hanging drop vapor diffusion method. Crystals were grown at 16°C by mixing 1 µl of the protein solution with 1 µl precipitant solution. Crystals of MRG2 (residues 41–108) in complex with H3K36me3 peptide were grown under conditions of 0.1 M HEPES pH 7.5, 12% PEG 6000, 5% MPD. Crystals of MRG2 (residue 53–123) in complex with H3K4me3/H3K36me3 were grown under conditions of 0.1 M MES pH 6.0, 27% PEG MME 5000, 0.2 M ammonium sulfate. Diffraction data were collected from flash-cooled crystals at 100K at SSRF (Shanghai Synchrotron Radiation Facility). The data was processed using HKL2000 [54]. Molecular-replacement solutions were generated using the Phaser in Phenix program and the crystal structure of MRG15 chromo domain (PDB entry code 2F5K) used as a search model. The initial models were built with COOT [55] and refined using Phenix [56]. The final refined structure was represented by Pymol (The PyMOL Molecular Graphics System, Version 1.4.1 Schrödinger, LLC). Coordinates have been deposited under PDB accession code 4PL6, 4PLI, and 4PLL.

## Supporting Information

Figure S1 MRG1 and MRG2 belong to the MRG protein family. Alignment of MRG1 and MRG2 chromodomain sequences with their homologs in *Saccharomyces cerevisiae* (Sc), *Drosophila melanogaster* (Dm), and *Homo sapiens* (Hs). The most conserved residues are highlighted in yellow, and relatively conserved residues are highlighted in green. Secondary structure elements are shown on the top. The missing helix in the structure is shown in a transparent color. Methylated peptide binding residues are labeled by stars.
(TIF)

Figure S2 ITC measurements of the binding between MRG2 chromodomain and histone peptides.
(TIF)

Figure S3 Flowering time analysis and relative expression levels of *CO* and *FT* in *35S::FLAG-CO* transgenic plants. Top panel, flowering times of indicated genotypes grown in LDs. The mean value from 20 plants is shown. Error bars represent standard deviations. Bottom panel, relative expression levels of *CO* and *FT* in indicated genotypes at ZT16. Values are presented as logarithmic mode (lg) of relative expression normalized to *ACTIN2*. Error bars show standard deviation from three replicates. A single asterisk indicates the statistically significant difference between *35S::FLAG-CO/mrg1 mrg2* plants and the wild-type (P<0.05), and double asterisks indicate the statistically significant difference between *35S::FLAG-CO/mrg1 mrg2* plants and *mrg1 mrg2* double mutants (P<0.05).
(TIF)

Figure S4 Relative expression levels of *MRG1* and *MRG2* in indicated genotypes. The wild-type (WT), *mrg1 mrg2* (*mrg1*(−/−) *mrg2*(−/−)) double mutant, transgenic *35S::MYC-CO* plants in wild-type (*35S::MYC-CO*), in heterozygous for *mrg1 mrg2* (*35S::MYC-CO/mrg1*(+/−) *mrg2*(+/−)), and in homozygous for *mrg1 mrg2* (*35S::MYC-CO/mrg1 mrg2*) were used for analyzed. Values are normalized to *ACTIN2*. Error bars show standard deviation from three replicates.
(TIF)

Figure S5 Relative expression levels of *MRG1/2* and H3K4me3/H3K36me3 levels at *FT* in wild-type (WT), and *co* plants at ZT16. A. Relative expression levels of *MRG1* and *MRG2* in indicated genotypes. Values are normalized to *ACTIN2*. Error bars show standard deviation from three replicates. B. ChIP analyses of H3K4 and H3K36 tri-methylation at *FT* chromatin in indicated genotypes at ZT16. Error bars show SD from three replicates.
(TIF)

Figure S6 Relative expression levels of *MRG1/2* and protein level of YFP-tagged MRG2 or MRG2(Y87A) in indicated genotypes at ZT16. A. Relative *MRG1* and *MRG2* levels in *35S::YFP* (*YFP*), *P_MRG2_::MRG2-YFP*/*mrg1 mrg2* (*MRG2-YFP*/*mrg1 mrg2*), and *P_MRG2_::MRG2(Y87A)-YFP*/*mrg1 mrg2* (*MRG2(Y87A)-YFP/mrg1 mrg2*) plants. Values are normalized to *ACTIN2*. Error bars show standard deviation from three replicates. B. Protein levels of YFP-tagged MRG2 or MRG2(Y87A) in indicated genotypes at ZT16.
(TIF)

Table S1 Crystallographic statistics.
(DOC)

Table S2 List of primers used in this study.
(DOC)
