# Supplementary figures and images for: Regulation of Arabidopsis Flowering by the Histone Mark Readers MRG1/2 via Interaction with CONSTANS to Modulate FT Expression
Source: PLoS Genet. 2014 Sep 11;10(9):e1004617. doi: 10.1371/journal.pgen.1004617 (PMC4161306; doi:10.1371/journal.pgen.1004617)

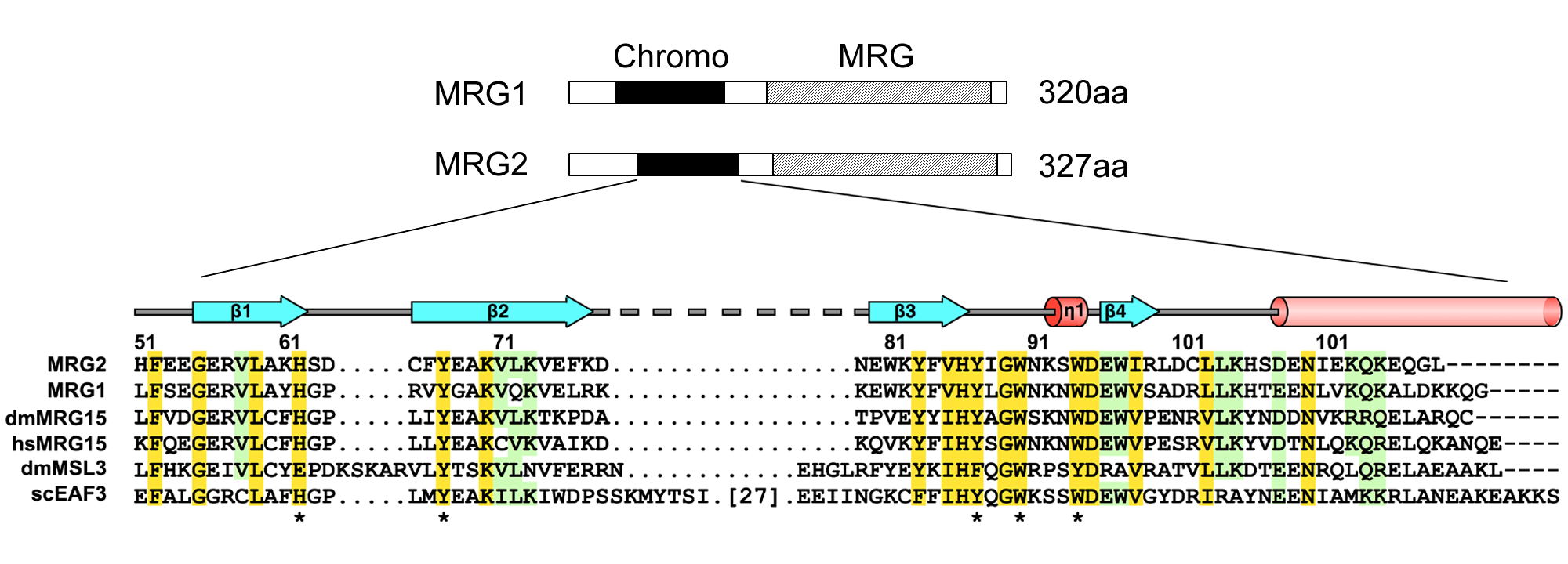

Supplement: Figure S1 — MRG1 and MRG2 belong to the MRG protein family. Alignment of MRG1 and MRG2 chromodomain sequences with their homologs in Saccharomyces cerevisiae (Sc), Drosophila melanogaster (Dm), and Homo sapiens (Hs). The most conserved residues are highlighted in yellow, and relatively conserved residues are highlighted in green. Secondary structure elements are shown on the top. The missing helix in the structure is shown in a transparent color. Methylated peptide binding residues are labeled by stars. (TIF) [file pgen.1004617.s001.tif]

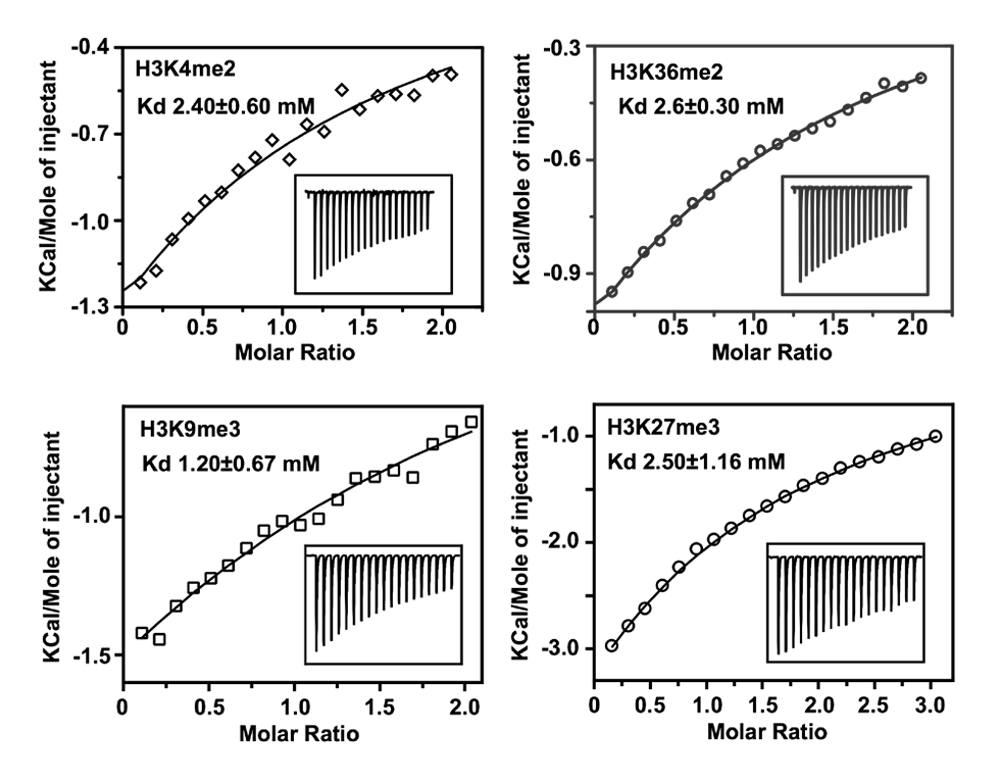

Supplement: Figure S2 — ITC measurements of the binding between MRG2 chromodomain and histone peptides. (TIF) [file pgen.1004617.s002.tif]

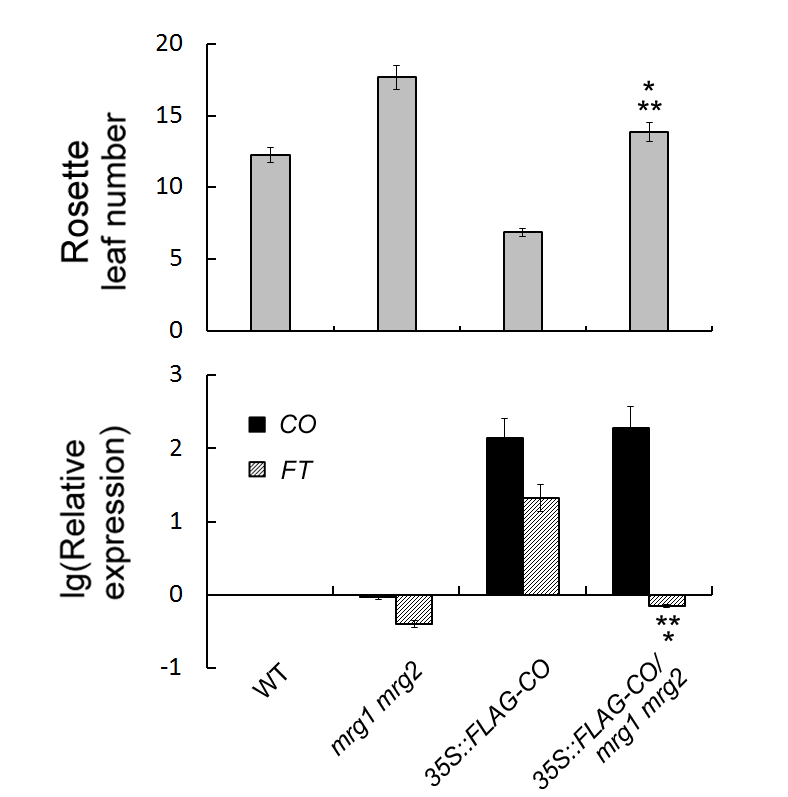

Supplement: Figure S3 — Flowering time analysis and relative expression levels of CO and FT in 35S::FLAG-CO transgenic plants. Top panel, flowering times of indicated genotypes grown in LDs. The mean value from 20 plants is shown. Error bars represent standard deviations. Bottom panel, relative expression levels of CO and FT in indicated genotypes at ZT16. Values are presented as logarithmic mode (lg) of relative expression normalized to ACTIN2. Error bars show standard deviation from three replicates. A single asterisk indicates the statistically significant difference between 35S::FLAG-CO/mrg1 mrg2 plants and the wild-type (P<0.05), and double asterisks indicate the statistically significant difference between 35S::FLAG-CO/mrg1 mrg2 plants and mrg1 mrg2 double mutants (P<0.05). (TIF) [file pgen.1004617.s003.tif]

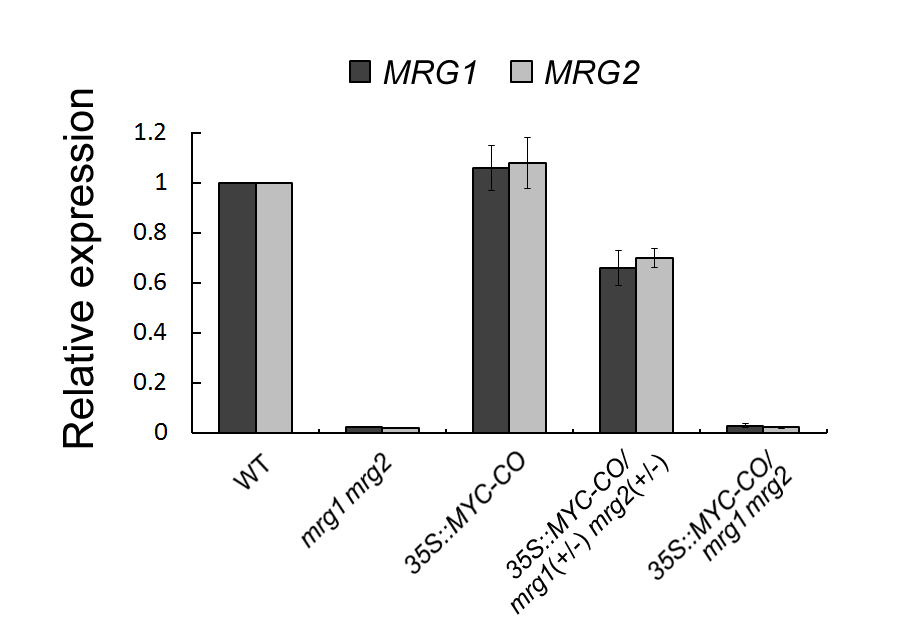

Supplement: Figure S4 — Relative expression levels of MRG1 and MRG2 in indicated genotypes. The wild-type (WT), mrg1 mrg2 (mrg1(−/−) mrg2(−/−)) double mutant, transgenic 35S::MYC-CO plants in wild-type (35S::MYC-CO), in heterozygous for mrg1 mrg2 (35S::MYC-CO/mrg1(+/−) mrg2(+/−)), and in homozygous for mrg1 mrg2 (35S::MYC-CO/mrg1 mrg2) were used for analyzed. Values are normalized to ACTIN2. Error bars show standard deviation from three replicates. (TIF) [file pgen.1004617.s004.tif]

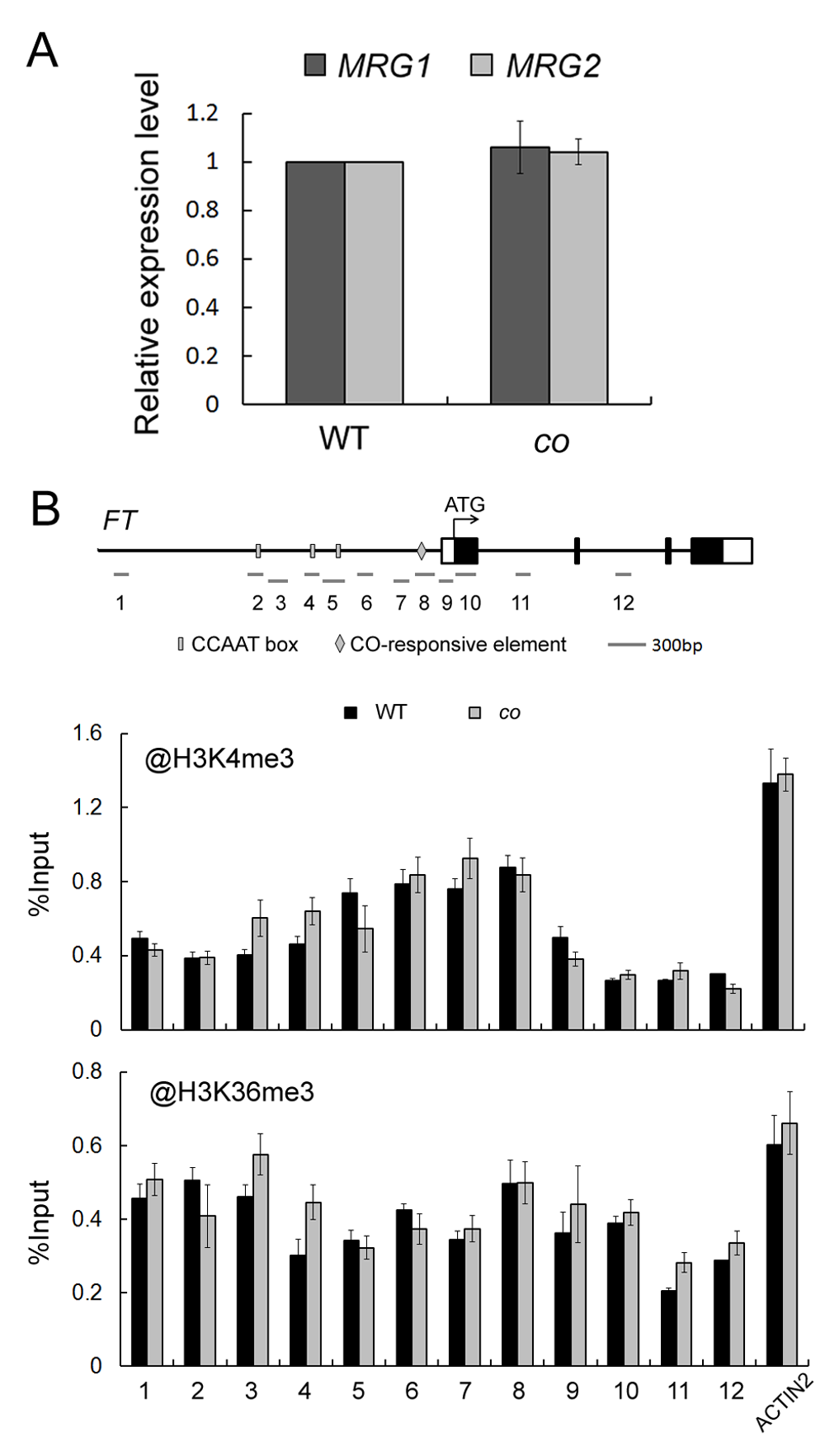

Supplement: Figure S5 — Relative expression levels of MRG1/2 and H3K4me3/H3K36me3 levels at FT in wild-type (WT), and co plants at ZT16. A. Relative expression levels of MRG1 and MRG2 in indicated genotypes. Values are normalized to ACTIN2. Error bars show standard deviation from three replicates. B. ChIP analyses of H3K4 and H3K36 tri-methylation at FT chromatin in indicated genotypes at ZT16. Error bars show SD from three replicates. (TIF) [file pgen.1004617.s005.tif]

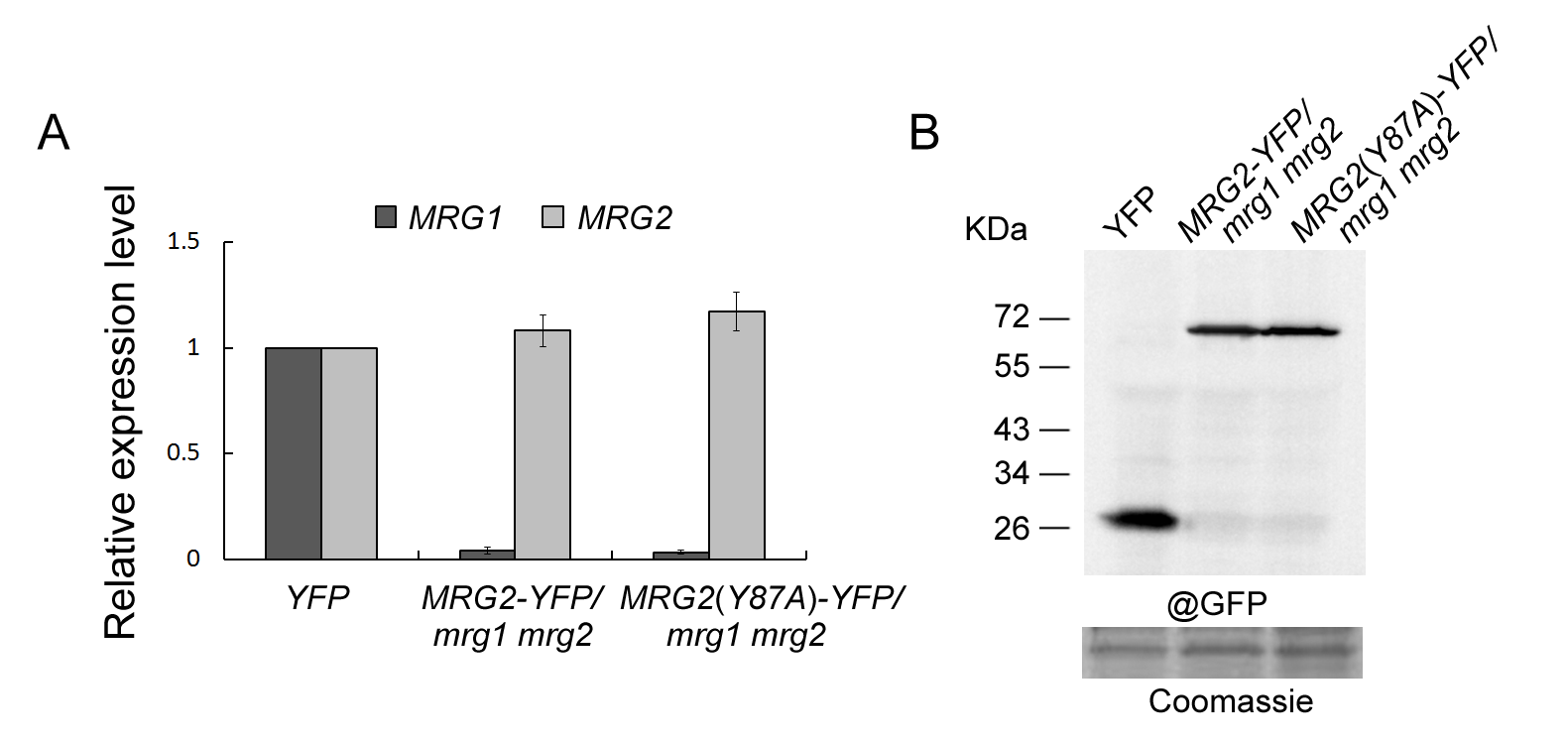

Supplement: Figure S6 — Relative expression levels of MRG1/2 and protein level of YFP-tagged MRG2 or MRG2(Y87A) in indicated genotypes at ZT16. A. Relative MRG1 and MRG2 levels in 35S::YFP (YFP), PMRG2::MRG2-YFP/mrg1 mrg2 (MRG2-YFP/mrg1 mrg2), and PMRG2::MRG2(Y87A)-YFP/mrg1 mrg2 (MRG2(Y87A)-YFP/mrg1 mrg2) plants. Values are normalized to ACTIN2. Error bars show standard deviation from three replicates. B. Protein levels of YFP-tagged MRG2 or MRG2(Y87A) in indicated genotypes at ZT16. (TIF) [file pgen.1004617.s006.tif]
