# Supplementary material for: Regulation of Arabidopsis Flowering by the Histone Mark Readers MRG1/2 via Interaction with CONSTANS to Modulate FT Expression
Source: PLoS Genet. 2014 Sep 11;10(9):e1004617. doi: 10.1371/journal.pgen.1004617 (PMC4161306; doi:10.1371/journal.pgen.1004617)
Supplement: Table S1 — Crystallographic statistics. (DOC) [file pgen.1004617.s007.doc]

**Table S1.** Crystallographic statistics

|  | MRG2 (53-123)  with H3K4me3 | MRG2 (41-108)  with H3K36me3 | MRG2 (53-123)  with H3K36me3 |
| --- | --- | --- | --- |
| **Data collection** |  |  |  |
| Space group | P6_5_ | P6_5_ | P6_1_ |
| Cell dimension |  |  |  |
| *a, b, c* (Å) | 54.2, 54.2, 127.9 | 54.3, 54.3, 127.8 | 109.2, 109.2, 30.0 |
| α, β, γ (°) | 90, 90, 120 | 90, 90, 120 | 90, 90, 120 |
| Wavelength (Å) | 0.9792 | 0.9787 | 0.9791 |
| Resolution range (Å)^a^ | 30.00-1.68 | 30.00-1.65 | 30.00-2.60 |
|  | (1.74-1.68) | (1.71-1.65) | (2.69-2.60) |
| Completeness (%)^a^ | 96.6 (93.6) | 98.6 (99.0) | 99.0 (97.4) |
| *R*_merge_ (%)^a^ | 5.8 (34.1) | 5.0 (43.8) | 9.4 (47.6) |
| *I* / σ*I* | 36.1 (2.2) | 47.2 (3.2) | 31.0 (2.4) |
| Redundancy | 3.3 (2.1) | 6.9 (4.4) | 6.7 (3.7) |
|  |  |  |  |
| **Refinement** |  |  |  |
| Resolution range (Å) | 25.0-1.68 | 25.0-1.65 | 28.6-2.6 |
| No. of reflections | 23,374 | 25,190 | 6,489 |
| *R*_work_ (%)/*R*_free_ (%) | 19.1/21.4 | 21.0/23.0 | 24.0/27.9 |
| R.m.s.deviations |  |  |  |
| Bond length (Å) | 0.006 | 0.007 | 0.01 |
| Bond angles (°) | 0.96 | 1.01 | 1.33 |
| Ramachandran plot |  |  |  |
| Most favored region (%) | 98.2 | 98.3 | 93.5 |
| Allowed region (%) | 1.8 | 1.7 | 5.6 |
| Outliers (%) | 0.0 | 0.0 | 0.9 |

^a^Values in parentheses are for the highest-resolution shell.
