# Supplementary material for: Regulation of Arabidopsis Flowering by the Histone Mark Readers MRG1/2 via Interaction with CONSTANS to Modulate FT Expression
Source: PLoS Genet. 2014 Sep 11;10(9):e1004617. doi: 10.1371/journal.pgen.1004617 (PMC4161306; doi:10.1371/journal.pgen.1004617)
Supplement: Table S2 — List of primers used in this study. (DOC) [file pgen.1004617.s008.doc]

**Table S2. List of primers used in this study.**

| Name | Sequence | Gene name |
| --- | --- | --- |
| Primers for constructs | | |
| MRG1-1 | 5'-aaggatccATGGGAAGCTCGTCGAAG-3' | *MRG1* |
| MRG1-2 | 5'-aactcgagTCAACCTTTTTCAACTCCTTGCT-3' |
| MRG1-3 | 5'-aactcgagAATCAATTTTTTTATATTTATTTGA-3' | *MRG1* |
| MRG1-4 | 5'-aaggatccGTCGTCTTTTCCTTTGCCTT-3' |
| MRG2-1 | 5'-aaggatccATGGGAAGCCCTAACGC-3' | *MRG2* |
| MRG2-2 | 5'-aactcgagCTACCCTTGCTGTTTCAAACC-3' |
| MRG2-3 | 5'-TGCATgcCATTGGTTGGAA-3' | *MRG2* |
| MRG2-4 | 5'-CAAAATATTTCCATTCATTGTCT-3' |
| MRG2-5 | 5'-aactcgagAATCAATTTTTTTATATTTATTTGA-3' | *MRG2* |
| MRG2-6 | 5'-aaggatccGTCGTCTTTTCCTTTGCCTT-3' |
| MRG2-7 | 5'-TGTGCATgcCATTGTAAGTAG-3' | *MRG2* |
| MRG2-8 | 5'-AAATATTTCCATTCATTGTCTTT-3' |
| MRG2-9 | 5'-aaggatccCGGAAGCAAGATTCTGTTGATAC-3' | *MRG2* |
| MRG2-10 | 5'-aactcgagCTAACCTTCTTTCTTCTCCATT-3' |
| MRG2-11 | 5'-aaggatccgcggactacaaagatgacgatgacaaaggaggaggctcaCGGAAGCAAGATTCTGTTGATAC-3' | *MRG2* |
| MRG2-12 | 5'-aagtcgactttgtcatcgtcatctttgtagtccgctgagcctcctccACCTTCTTTCTTCTCCATT-3' |
| CO-1 | 5'-aaggatccATGTTGAAACAAGAGAGTAACGAC-3' | *CO* |
| CO-2 | 5'-aagtcgacTCAGAATGAAGGAACAATCCC-3' |
| CO-3 | 5'-aaggatccgcggactacaaagatgacgatgacaaaggaggaggctcaATGTTGAAACAAGAGAGTAACGAC-3' | *CO* |
| CO-4 | 5'-aagtcgactttgtcatcgtcatctttgtagtccgctgagcctcctccGAATGAAGGAACAATCCC-3' |
| Primers for RT-PCR | | |
| ACTIN2-F | 5'-AGTGTTAGCTGCTGCCGCTGT-3' | *ACTIN2* |
| ACTIN2-R | 5'-ACCAGCAAAACCAGCCTTCACCA-3' |
| MRG1-F | 5'-CCTCGTTCACCTAACGTCGATGAGA-3' | *MRG1* |
| MRG1-R | 5'-GGCAATGCCTTGTCGAAATAACTCC-3' |
| MRG2-F | 5'-TTGACAGCCAAATGAAGAAACATGG-3' | *MRG2* |
| MRG2-R | 5'-CCCCCTGATACGGATTCCTCGTA-3' |
| CO-F | 5'-CAGGGACTCACTACAACGACAATGG-3' | *CO* |
| CO-R | 5'-GGTTGTTGCTCTACTGTCCCTTTGG-3' |
| FT-F | 5'-TGGTGGATCCAGATGTTCCAAGTC-3' | *FT* |
| FT-R | 5'-CTCATTGCCAAAGGTTGTTCCAGTT-3' |
| Primers for ChIP-PCR | | |
| FT-1-F | 5'-GCATGCGAAAATCTAGTGGAAGA-3' | *FT* |
| FT-1-R | 5'-GTCGCATAATGTTCGCAACCT-3' |
| FT-2-F | 5'-GCGACTGCGACCTATTTTTTTC-3' | *FT* |
| FT-2-R | 5'-GCTATATGCACTTTTTAACGACTAGC-3' |
| FT-3-F | 5'-GCTAGTCGTTAAAAAGTGCATATAG-3' | *FT* |
| FT-3-R | 5'-CCACTGTTCTACACGTCCATAG-3' |
| FT-4-F | 5'-TCCTTTATTTTCCAGTTTGGACAG-3' | *FT* |
| FT-4-R | 5'-ACCATAGCCTAACAACTGTAGGAA-3' |
| FT-5-F | 5'-CCTACAGTTGTTAGGCTATGGT-3' | *FT* |
| FT-5-R | 5'-CTAACCATCCATTTGCACGAC-3' |
| FT-6-F | 5'-TCTAACCTGAAGGATCCCTTG-3' | *FT* |
| FT-6-R | 5'-AATTCGAAAGCGAAAACGTTC-3' |
| FT-7-F | 5'-GAACGTTTTCGCTTTCGAATT-3' | *FT* |
| FT-7-R | 5'-GAAAAAAGTAGGGTACCGCC-3' |
| FT-8-F | 5'-AGTGTGGTGGGTTTGGAATAC-3' | *FT* |
| FT-8-R | 5'-GCATTAACTCGGGTCGGTGA-3' |
| FT-9-F | 5'-AGAGGGTTCATGCCTATGATAC-3' | *FT* |
| FT-9-R | 5'-CTTTGATCTTGAACAAACAGGTG-3' |
| FT-10-F | 5'-GAGACCCTCTTATAGTAAGCAGA-3' | *FT* |
| FT-10-R | 5'-GTATAGAAGTTCCTGAGGTCTTC-3' |
| FT-11-F | 5'-GACAATGTGTGATGTACGTAGAATCAGT-3' | *FT* |
| FT-11-R | 5'-CGGTGAAATCATAACCACAATCTT-3' |
| FT-12-F | 5'-GCCAGCCTTTAAGATACTCTCTGCTA-3' | *FT* |
| FT-12-R | 5'-TGAGATAACACAAGAAAGAAGAAGAAAACT-3' |
| ACT2-F | 5'-GATGAGGCAGGTCCAGGAATC-3' | *ACTIN2* |
| ACT2-R | 5'-AACCCCAGCTTTTTAAGCCTTT-3' |
